# Physical development and sociocultural context influences on children’s physical activity

**DOI:** 10.1590/1984-0462/2025/43/2024113

**Published:** 2025-01-20

**Authors:** Alessandro Hervaldo Nicolai Ré, Maria Teresa Cattuzzo, David Stodden, Guilherme dos Santos, Albert Lucas Olinto Tertuliano, Carlos Bandeira de Mello Monteiro, Diogo Henrique Constantino Coledam, Anthony David Okely

**Affiliations:** a Universidade de São Paulo, São Paulo, SP, Brazil.; b Universidade de Pernambuco, Recife, PE, Brazil.; c University of South Carolina, Columbia, SC, United States of America.; d Instituto Federal de Educação, São Paulo, SP, Brazil.; e University of Wollongong, Wollongong, NSW, Australia.

**Keywords:** Motor skills, Physical fitness, Health promotion, Child development, Socioeconomic factors, Habilidades motoras, Aptidão física, Promoção da saúde, Desenvolvimento infantil, Fatores socioeconômicos

## Abstract

**Objective:**

To examine the predictive strength of cardiorespiratory fitness (CRF), motor competence (MC), maternal educational status, and parental perception of their children’s favorite leisure activities for meeting physical activity (PA) guidelines of each gender among children aged 3–6 years.

**Methods:**

This is a cross-sectional study with 367 preschoolers (53% girls), assessing CRF (PREFIT 20m shuttle run), MC (battery of motor skills), PA (accelerometry), maternal education and parental perception of children’s preferred leisure (questionnaires). Factorial analysis of covariance, multiple logistic regression, and chi-square tests were conducted.

**Results:**

In both genders, CRF was associated with meeting PA guidelines (girls: odds ratio [OR] 1.10; 95% confidence interval [CI] 1.03–1.18; boys: OR 1.12; 95%CI 1.05–1.19), independently of maternal education or parental perception of children’s leisure activities. For girls, active leisure with balls (OR 28.91; 95%CI 6.88–121.50) and without balls (OR 4.32; 95%CI 1.95–9.57) enhanced the odds of compliance with PA guidelines, without effect of maternal education. For boys, maternal education was inversely associated with meeting the PA guidelines. Boys of lower-educated mothers were more likely to have activities with balls as favorite leisure, which was a stronger predictor and enhanced the odds of meeting the guidelines (OR 4.09; 95%CI 1.71–9.79) regardless of maternal education. Boys had a higher prevalence of active leisure with balls than girls (42.8 vs. 7.7%).

**Conclusions:**

Regardless of CRF or MC, family and sociocultural circumstances influenced PA according to gender. Health policies should encourage equal gender participation in culturally significant sports or activities of a particular region/country, specifically within the family context.

## RESUMO

**Objetivo:**

Examinar a força preditiva da aptidão cardiorrespiratória (ACR), competência motora (CM), nível educacional materno e percepção dos pais sobre as atividades de lazer preferidas dos filhos, para atender as diretrizes de atividade física (AF) de cada gênero, dos 3 aos 6 anos.

**Métodos:**

Este é um estudo transversal com 367 pré-escolares (53% meninas), testando ACR (teste PREFIT vaivém 20 m), CM (bateria de habilidades motoras), AF (acelerometria), nível educacional materno e percepção dos pais sobre o lazer preferido dos filhos (questionários). Foram realizados os testes de análise de covariância fatorial, regressão logística múltipla e qui-quadrado.

**Resultados:**

Em ambos os gêneros, ACR esteve associada ao cumprimento das diretrizes de AF (meninas: razão de probabilidade [OR] 1,10; intervalo de confiança [IC95%] 1,03–1,18; menino: OR 1,12; IC95% 1,05–1,19), independentemente da escolaridade materna ou da percepção dos pais sobre o lazer dos filhos. Para as meninas, o lazer ativo com bola (OR 28,91; IC95% 6,88–121,50) e sem bola (OR 4,32; IC95% 1,95–9,57) aumentou as chances de atendimento das diretrizes de AF, sem efeito da escolaridade materna. Para os meninos, a escolaridade materna foi inversamente associada ao cumprimento das diretrizes de AF. Os meninos de mães com menor escolaridade eram mais propensos a ter atividades com bolas como lazer favorito, o que foi um preditor mais forte e aumentou as chances de atender às diretrizes (OR 4,09; IC95% 1,71–9,79), independentemente da situação educacional materna. Os meninos apresentaram maior prevalência de lazer ativo com bola do que as meninas (42,8% vs. 7,7%).

**Conclusões:**

Independentemente da ACR ou da CM, circunstâncias familiares e socioculturais influenciaram a AF conforme o gênero. As políticas de saúde devem encorajar a participação igualitária de gênero em atividades/esportes culturalmente significativos em determinada região/país, especificamente no contexto familiar.

## INTRODUCTION

Physical activity (PA) guidelines suggest preschoolers accumulate at least 180 min of PA daily with at least 60 min of moderate-to-vigorous intensity (MVPA).^1^ Despite the reported associations between PA and health indicators in the early years,^1^ inadequate PA levels in childhood remain a public health problem.^2^ Preschoolers with low levels of PA tend to increase their levels of physical inactivity during late childhood and adolescence, which further restricts motor development and can generate a negative behavioral cycle, contributing to the emergence of chronic diseases such as diabetes, hypertension, and obesity,^2^ in addition to potential negative impacts on cognitive and psychosocial development.^1^ Recent data demonstrate a decline in cardiorespiratory fitness (CRF) and motor competence (MC) (proficiency in motor skills, performed with control and coordination) across childhood.^3,4,5^ Both low CRF and poor MC have been associated with reduced PA levels and increased sedentary behavior.^3,6^

In regions of high social vulnerability, factors such as violence, school failure, and lack of appropriate space and equipment increase the likelihood of physical inactivity and delays in motor development.^7,8,9,10^ Children’s home environments may act as facilitators for PA since many opportunities may be determined by the youth’s home circumstances, rather than the wider environment in which they live.^11^ Specifically, maternal education and leisure activities provided by the family may impact children’s PA and the development of CRF and MC.^11,12^ Improved parental education, particularly of mothers, is linked to increased access to health information, which could promote positive experiences for children’s healthy development.^13^

However, there is inconsistent evidence on the association between parental education and children’s PA,^14,15,16^ partly due to the multifaceted influences of demographic and sociocultural factors, such as gender, marital status, birth order, time spent outdoors, and leisure opportunities.^5,17^ While positive associations between CRF, MC, and PA have generally been reported,^18,19^ no known study has examined the combined influence of maternal education and lifestyle behaviors (leisure activities) that may influence PA and motor performance of girls and boys. This knowledge could help researchers, policymakers, and practitioners to improve health promotion strategies and social policies. Thus, the aim of this study was to examine the strength of CRF, MC, maternal education status, and parents’ perception of their child’s favorite leisure activities for meeting PA guidelines for each gender among children aged 3–6 years.

## METHOD

This cross-sectional study included a population of 3,000 preschool children aged 3–6 years (mean [M] 4.9, standard deviation ±0.6 years; 47% girls) from four preschools in Eastern São Paulo, Brazil. For the sample size calculation, a total of 341 children was required, assuming an estimated prevalence of 50% for the outcome, a tolerable error of 5%, and a confidence level of 95%. Data were collected over seven years (2013–2019). Children generally stayed in preschool for three years, with data collection focused on those aged 3–6. Considering this age range and ensuring that no child was counted more than once, the total number of children during the study period was approximately 3000, with an average of 428 new children per academic year.

Schools were selected through convenience sampling, with the inclusion criterion being located in socially vulnerable communities in São Paulo city. Postcodes of schools and family residences identified the area of living. The social vulnerability map of São Paulo, which considers access to public services, indicators of urban violence, and economic deprivation of families was used to identify areas of high social vulnerability. The Ethics Committee of the University of São Paulo approved this study (CAEE 24766913.0.0000.5390). The study complied with the guidelines of resolution 466/2012. Children who provided assent, returned signed parental consent, and had no medical conditions preventing PA (reported by parents and/or a doctor) were included in the data collection (n=2234).

The final sample considered 367 children with complete data (mean age 4.8±0.6 years). The remaining 1867 children with partial data were excluded from the main analyses but were used to assess selection bias, confirming sample representativeness. No imputation or data modeling was used to replace missing data, and data for each child were collected within eight weeks to avoid the influence of biological development.

Accelerometers (ActiGraph GT3X+, Pensacola, FL) assessed PA, with data processed in ActiLife software version 6 (ActiGraph Pensacola, FL). Activities were recorded in 15-s epoch at 30 Hz. Participants wore the accelerometer for seven consecutive days. The non-wear time was defined as 60 min of consecutive zeros allowing for 2 min of non-zero interruptions. A valid day required 500 min of wear time, between 6 a.m. and 11 p.m. This threshold ensured the accuracy and reliability of PA measurements.^20^ Participants with valid data for at least three days (considering at least one weekend day) were included. Evenson et al. cut-points defined daily minutes and the percentage of wear time spent in sedentary (<100 cpm), light (101–2295 cpm), moderate (2296–4011 cpm), and vigorous (≥4012 cpm) PA.^21^ The mean daily MVPA minutes were used to identify children meeting the PA guidelines (≥60 min/day MVPA).^1^

The PREFIT 20 m shuttle run, a reliable tool for preschoolers, evaluated CRF.^22^ Participants ran back and forth between two lines 20 meters apart, starting at 6.5 km/h, with increments of 0.5 km/h each minute. Two evaluators ran with groups of 3–6 children to maintain pace and provide motivation. An audio recording indicated the pace, and the test finished when the child missed two consecutive audio signals or voluntarily stopped due to fatigue. The number of completed laps was recorded.

Fundamental motor skill competence was assessed using six skills from the Test of Gross Motor Development 2 (TGMD-2).^23^ This reduced version of TGMD-2 was used due to time constraints and because compilations of different skills provide an adequate overall measure of MC compared to all 12 skills.^24^ Children’s performances were video-recorded and three locomotor (hop, horizontal jump, and slide: total raw score 0–26) and three object control (kick, catch, and overhand throw: total raw score 0–22) skills were included. Two trained researchers with prior experience (at least 20 hours) in analyzing TGMD-2 separately coded the data. Disagreements were reassessed by both raters to define the final value (0 or 1), resulting in 100% agreement between them. Inter- and intra-rater reliability was checked using the percent agreement method [number of agreements/(number of agreements+disagreements)×100] based on videos of 20 children who completed the six skills. Mean inter-observer agreement was 90.7% (85.2–96.7%) and intra-rater agreement was 92.1% (86.1–98.4%).

Parents/guardians completed a survey providing sociodemographic information about the child and family, including parental age, education, marital status, birth order, household composition, socioeconomic status, and the child’s main leisure activity. Socioeconomic status was quantified using the Brazilian Economic Classification Criteria.^25^ Parental education was dichotomized as having or not having completed high school (i.e., the lower-educated group did not complete high school).

Parents also answered a question about their perception of their child’s favorite leisure activities that was: “What is your child’s favorite leisure activity?”

This open-ended question^26^ was categorized into three groups:

a) sedentary leisure activities, which included screen activities (TV, videogames, etc.) and low energetic play like drawing, painting, playing with dolls, mini-cars, and symbolic games like cooking;
b) active leisure without balls such as dancing, running, cycling, going to parks; and
c) active leisure with balls (e.g., soccer and “playing with a ball”).

The reliability of parents’ responses was checked using the percent agreement method, based on 37 parents who answered the same question twice, 60–80 days apart. The mean percentage of agreement was 94.6%.

Analyses were conducted separately by gender using Statistical Package for Social Sciences (SPSS) version 26.0 with a significance level of p<0.05. All necessary assumptions for the statistical tests were met. A factorial analysis of covariance (ANCOVA) [leisure activities (3) × maternal education (2)] examined the effects of maternal education and leisure activities on the dependent variables, adjusted for age, school, and accelerometer wear time, with Tukey’s post-hoc test. Effect size was estimated using partial eta squared (η^2^) and interpreted as small (0.01), medium (0.06), or large (0.14). Pearson’s correlation coefficients (r) were also calculated.

Multiple logistic regression models adjusted for age, school, and accelerometer wear time, assessed the predictive power of CRF (PREFIT laps), MC, body mass index, and each sociodemographic variable (parental age, education, marital status, birth order, household composition, socioeconomic status, and parents’ perception of their child’s favorite leisure activities) for meeting the PA guidelines (data not shown). Variables with statistically significant coefficients (p<0.05) were retained for the main analysis. Thus, logistic regression models evaluated the predictive utility of CRF, MC, maternal education, and parents’ perception of their child’s favorite leisure activities for meeting PA guidelines. To test the strength of the predictors, three logistic regression models were used: first considering CRF and MC (model 1), followed by the inclusion of maternal education (model 2), and then parents’ perception of their child’s favorite leisure activities (model 3). Chi-square tests (χ^2^) were used to assess the bivariate associations between maternal education, parental perception of leisure activities, meeting the PA guidelines, and gender.

## RESULTS

**Figure 1:**
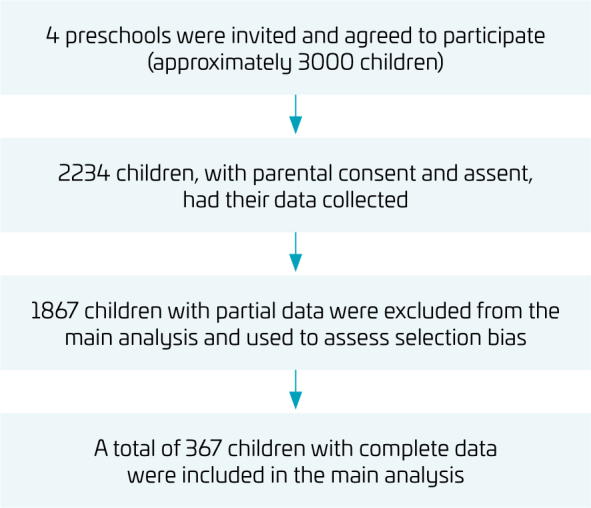
Flowchart showing data-collection and analysis procedure.

Approximately 3000 children aged 3–6 years were enrolled in four preschools. Of these, 2234 participated in the data collection. From this group, 1867 children had partial data (partial data group) and were used to assess selection bias, while 367 children had complete data (study sample) and were included in the main analyses (Figure 1). In preliminary analyses (Table 1), one-way analysis of variance (ANOVA) was used for continuous variables (age, CRF, MC, MVPA, and vigorous PA), and chi-square tests were performed for categorical variables (maternal education, leisure activities, and the proportion of children meeting PA guidelines) to compare the study sample (n=367) with the partial data group (n=1867). No statistically significant differences were found between these groups for any variable (p>0.05) and all subsequent analyses were conducted using only children with complete data (study sample).

**Table 1:** Comparisons between study sample and partial data group by gender.

Gender (sample, n) Age (years) M±SD	CRF SR, laps M±SD	OC, raw score M±SD	LOC, raw score M±SD	MVPA, minutes M±SD)	Maternal Education		Parents’ perception about child’s leisure activities			Meeting PA guidelines n (%)
					Low n (%)	High n (%)	SA n (%)	Active without balls n (%)	Active with balls n (%)	
Girls (study sample, n=194)										
4.8±0.6	10.8±5.3	11.5±2.5	15.2±3.9	50.3±17.3	79 (40.7)	115 (59.3)	128 (66.0)	51 (26.3)	15 (7.7)	53 (27.3)
Girls (partial data group, n=845)										
4.8±0.6	n=19 11.1±8.8	n=636 11.4±2.3	n=636 15.0±3.1	n=153 52.1±17.4	73 (42.7)	98 (57.3)	120 (62.8)	58 (30.4)	13 (6.8)	47 (30.7)
Boys (study sample n=173)										
4.9±0.6	13.9±7.4	12.8±2.8	15.3±4.4	60.7±21.3	85 (49.1)	88 (50.9)	51 (29.5)	48 (27.7)	74 (42.8)	86 (49.7)
Boys (partial data group, n=1021)										
5.0±0.6	n=52 14.4±9.1	n=761 12.6±2.8	n=760 14.9±3.4	n=205 63.5±22.6	86 (48.3)	92 (51.7)	65 (32.8)	69 (34.8)	64 (32.3)	112 (54.6)

M±SD: mean ± standard deviation; n (%): absolute and relative frequencies; CRF SR: cardiorespiratory fitness 20 m shuttle run; OC: object control skills; LOC: locomotor skills; MVPA: moderate to vigorous physical activity (daily minutes); Maternal education: low = did not complete high school, high = completed high school or superior degree; SA: sedentary activities; PA: physical activity.

Table 2 presents descriptive data and group comparisons by maternal education and parent-reported leisure activities. The ANCOVA results (p<0.05) for both genders, revealed a significant effect of maternal education on object control (OC) and locomotor skills (LOC), and a significant effect of leisure activities on PA, with higher values among children of less-educated mothers (OC and LOC), and those engaged in “leisure with balls” (MVPA and %VPA). Pearson’s correlation coefficients showed weak or non-existent associations between CRF and MC with continuous PA measures (MVPA, mean daily sedentary minutes, and % of vigorous PA) in both genders (r=0.01–0.29; data not shown).

**Table 2:** Mean values (standard deviation) and comparisons between groups of girls and boys with different maternal education and parents’ perception of leisure activities.

Factor/Level	n	PREFIT SR, laps Mean±SD	Object Control, raw score Mean±SD	Locomotor, raw score Mean±SD	MVPA, daily minutes Mean±SD	Vigorous PA, percentage Mean±SD
Girls						
Maternal education						
Less than high school	79	10.4±4.8	11.8±2.6*	16.0±3.8*	51.5±18.1	2.0±1.1
High school or higher	115	11.0±5.7	11.3±2.4*	14.7±3.9*	49.5±16.7	1.7±0.9
η^2^		0.001	0.053	0.043	0.000	0.008
Leisure group (parent’s perception)						
Active with balls	15	11.7±4.5	12.6±2.8	15.1±4.1	72.5±17.3^†,‡,§^	3.0±1.3^†,‡,§^
Active without balls	51	10.9±4.5	11.5±2.4	15.4±2.7	54.4±20.2^†,§,//^	2.0±1.1^†,§^
Sedentary	128	10.6±5.7	11.3±2.5	15.2±4.3	46.1±13.2^†,‡,//^	1.6±0.8^†,‡^
η^2^		0.006	0.016	0.005	0.179	0.128
Total	194	10.8±5.3	11.5±2.5	15.2±3.9	50.3±17.3	1.8±1.0
Boys						
Maternal education						
Less than high school	85	14.7±8.1	13.7±3.0*	16.3±4.4*	64.8±22.2	2.7±1.5
High school or higher	88	13.1±6.7	11.9±2.3*	14.3±4.2*	56.7±19.6	2.2±1.3
η^2^		0.003	0.082	0.038	0.004	0.013
Leisure group (parent’s perception)						
Active with balls	74	14.6±8.4	13.3±2.6	15.8±4.1	66.8±22.0^†,‡^	2.7±1.5^†,‡^
Active without balls	48	14.2±6.6	12.4±2.6	14.9±4.6	59.2±18.6	2.6±1.4
Sedentary	51	12.7±6.8	12.4±3.3	14.9±4.6	53.3±20.3^†,‡^	2.0±1.1^†,‡^
η^2^		0.004	0.006	0.001	0.063	0.043
Total	173	13.9±7.4	12.8±2.8	15.3±4.4	60.7±21.3	2.5±1.4

*p<0.05: difference between groups based on maternal education, within the same gender;

^†^p<0.05: difference between groups based on leisure group, within the same gender (post-hoc Tukey);

^‡^active with balls vs. sedentary activities;

^§^active with balls *vs*. active without balls;

^//^active without balls vs. sedentary activities.

SD: standard deviation; MVPA: moderate to vigorous physical activity; PA: physical activity; η^2^ partial eta squared.

Regardless of maternal education or leisure activities, CRF (PREFIT laps) was associated with meeting PA guidelines for both girls (OR 1.10; 95%CI 1.03–1.18) and boys (OR 1.12; 95%CI 1.05–1.19) (Table 3, model 3). Maternal lower education increased the odds of meeting PA guidelines in boys (OR 2.49; 95%CI 1.23–5.03) but not in girls (p=0.57) (Table 3, model 2). In both genders, parents’ perception of leisure activities, particularly with the ball (girls, OR 28.91; 95%CI 6.88–121.50; boys, OR 4.09; 95%CI 1.71–9.79), was associated with meeting the guidelines (Table 3, model 3). For boys, when parents’ perception of leisure activities was included among the predictors (Table 3, model 3), the association between maternal education and PA was suppressed, indicating that perception of leisure activity with the ball was a stronger predictor of PA, independent of maternal education.

**Table 3:** Logistic regression models for predicting the compliance with physical activity guidelines among girls and boys.

	Model 1			Model 2			Model 3		
	OR	LCI	UCI	OR	LCI	UCI	OR	LCI	UCI
Girls, n=194									
PREFIT SR, laps	1.09*	1.02	1.16	1.09*	1.02	1.16	1.10*	1.03	1.18
Object Control (raw score)	0.99	0.85	1.14	0.98	0.85	1.14	0.91	0.76	1.07
Locomotor (raw score)	1.05	0.96	1.16	1.05	0.96	1.16	1.08	0.96	1.21
Maternal education (low)				1.22	0.62	2.43	0.92	0.42	2.01
Sedentary leisure – Ref.									
Active leisure with balls							28.91*	6.88	121.50
Active leisure without balls							4.32*	1.95	9.57
Boys, n=173									
PREFIT SR, laps	1.11*	1.05	1.17	1.11*	1.05	1.17	1.12*	1.05	1.19
Object Control (raw score)	1.12	0.97	1.29	1.07	0.92	1.25	1.08	0.92	1.26
Locomotor (raw score)	0.95	0.86	1.04	0.94	0.85	1.03	0.94	0.85	1.03
Maternal education (low)				2.49*	1.23	5.03	1.82	0.86	3.84
Sedentary leisure – Ref.									
Active leisure with balls							4.09*	1.71	9.79
Active leisure without balls							2.28	0.93	5.60

OR: odds ratio; LCI: lower confidence interval; UCI: upper confidence interval; Ref: reference.

*p<0.05

Boys with lower-educated mothers had significantly more active leisure with balls in parental perceptions (χ^2^=19.07; p<0.001; Table 4), which increased the odds of meeting the guidelines. In girls, there were no associations between maternal education and parents’ perception of leisure activities (p=0.11; Table 4). There was a significant association between gender and leisure activities, with parents’ perception indicating that boys participated in fewer sedentary leisure activities and more active leisure activities with the ball than girls (χ^2^=71.36; p<0.001; Table 4). There was also a significant association between gender and meeting the PA guidelines (χ^2^=19.49; p<0.001; Table 4).

**Table 4:** Association between maternal education and leisure activities in girls and boys: total study sample and children meeting physical activity guidelines.

Maternal education	Leisure activity, n (%)			Total
	Sedentary	Active without balls	Active with balls	
Girls*				
Less than high school	46 (23.7)	24 (12.4)	9 (4.6)	79 (40.7)
Meeting PA guidelines^†^	6 (13.0)	9 (37.5)	8 (88.9)	23 (29.1)
High school or higher	82 (42.3)	27 (13.9)	6 (3.1)	115 (59.3)
Meeting PA guidelines^†^	14 (17.1)	12 (44.4)	4 (66.7)	30 (26.1)
Total^‡^	128 (66.0)	51 (26.3)	15 (7.7)	194 (100)
Meeting PA guidelines^§^	20 (15.6)	21 (41.2)	12 (80.0)	53 (27.3)
Boys*				
Less than high school	15 (8.7)	20 (11.6)	50 (28.9)	85 (49.1)
Meeting PA guidelines^†^	1 (6.7)	13 (65.0)	38 (76.0)	52 (61.2)
High school or higher	36 (20.8)	28 (16.2)	24 (13.9)	88 (50.9)
Meeting PA guidelines^†^	13 (36.1)	11 (39.3)	10 (41.7)	34 (38.6)
Total^‡^	51 (29.5)	48 (27.7)	74 (42.8)	173 (100)
Meeting PA guidelines^§^	14 (27.5)	24 (50.0)	48 (64.9)	86 (49.7)

PA: physical activity.

*Association between maternal education and leisure activity (stratified by gender): Chi-square tests (χ^2^): for girls χ^2^=4.37, p=0.112; for boys χ^2^=19.07, p<0.000;

^†^Association between maternal education and leisure activity within children physical activity guidelines (stratified by gender): Chi-square tests (χ^2^) for girls χ^2^=4.11, p=0.128; for boys χ^2^=24.07, p<0.001;

^‡^Association between gender and leisure activity: Chi-square tests (χ^2^) χ^2^=71.36, p<0.000;

^§^Association between gender and meeting physical activity guidelines: Chi-square tests (χ^2^): χ^2^ = 19.49, p<0.001.

## DISCUSSION

This study investigated the predictive utility of CRF, MC, maternal education, and parents’ perception of their child’s favorite leisure activities for meeting PA guidelines for each gender and tested the strength of these predictors on the associations with PA. In both genders, CRF (but not MC) was positively associated with meeting the PA guidelines, regardless of maternal education or parents’ perception of leisure activities. Other studies have shown low to moderate associations between CRF, MC, and PA.^5,18,19^ Maternal education was inversely associated with meeting the PA guidelines for boys, but not for girls, suggesting that gender may moderate the associations between these variables. Supporting this view, for both boys and girls, parents’ perception of active leisure activities was a strong predictor of meeting PA guidelines, but for girls, there was no association between maternal education and leisure activities. Most parents reported that girls participated in sedentary leisure activities, regardless of maternal education. For boys, the perception of active leisure with balls was a stronger predictor than maternal education for meeting the PA guidelines. Boys of lower-educated mothers were more likely to participate in active leisure with balls.

Cultural values and beliefs have been linked to differences between girls and boys in PA, fitness, and MC levels.^3^ In this study, there was a significant association between leisure activities and gender (p<0.001), with girls engaging in more sedentary leisure activities (66% vs. 29%) and less active leisure with balls (8% vs. 43%, respectively) than boys. The probable cultural belief that promotes less energetic play for girls, particularly in activities involving balls/soccer, tends to be consistent across all social classes, regardless of maternal education.^27^ Interestingly, potentially greater access to health-related information (e.g., through higher education) did not favor the choices for active leisure or PA.

An explanation for these results may be related to a strong cultural appeal, and the consequent increased motivation and opportunities in Brazil for boys to practice soccer and/or ball skills. In addition, having access to a ball may be the most accessible and cost-effective leisure activity for children of lower-educated mothers. From a public health perspective, ball skills seem to be a strong indicator of engagement and maintenance of PA and health-related fitness across childhood and into adolescence,^28^ requiring the development of complex coordination and control, which are integrated with locomotor and stability skills. Within this context, the promotion of ball games (e.g., soccer, basketball), particularly for girls, should be emphasized, especially because active leisure with balls was the main predictor of PA in both genders.

However, greater attention to motor development is needed, even among physically active children. In this study, although parent-reported active leisure activities were significant predictors of PA, there were no differences in CRF and MC between the groups with different leisure activities (sedentary leisure, active leisure without balls, and active leisure with balls), suggesting that increased PA in the active leisure groups did not consistently result in the development of CRF or MC. Possibly, the social vulnerability associated with this sample may limit children’s opportunities for instruction and practice of different types of PA in appropriate learning settings.^5,7,8^ The lack of developmentally appropriate motor learning opportunities could impact the quality of PA and the development of CRF and MC,^29^ even among children with high levels of PA.^5^

This study’s limitations include its cross-sectional design, which limits causal inferences, and the lack of detailed information on the quality and type of PA. Although parents’ perception of a child’s main leisure activity was based on only one question, its simplicity is noteworthy, as it may serve as a potentially important predictor of children’s PA. Its relevance to current societal trends, particularly the rise in sedentary activities linked to technology use, could help identify children at higher risk of inactivity in schools from a public health perspective. This is the first study to examine the combined influences of CRF, MC, and socioecological variables in meeting the PA guidelines in a disadvantaged area of a large urban center in Brazil, offering insights for future research in similar environments globally.

In conclusion, family and sociocultural circumstances influenced PA regardless of CRF or MC. Gender moderated the associations between maternal education, leisure, and PA. For girls, active leisure was a strong predictor of meeting PA guidelines, but there was no association with maternal education. For boys, the inverse association between maternal education and meeting PA guidelines was completely suppressed by active leisure with balls. Boys engaged in more active leisure than girls, particularly with balls (soccer). These results may be explained by the fact that soccer is rooted in Brazilian culture, which favors boys’ practice, and ball is a low-cost toy accessible to lower-income families.

This study highlights the need for equal PA opportunities for girls and boys, including the promotion of culturally valued sports for both genders as a key public health strategy. It is essential to improve knowledge related to physical development and PA among both parents and preschool teachers and to provide preschools and communities with adequate space and equipment to promote more diverse opportunities for health-enhancing PA. Longitudinal studies on leisure and family circumstances affecting childhood PA are needed to inform public health policies, particularly in underserved communities.

## Data Availability

The database that originated the article is available with the corresponding author. CAAE: 55476621.7.0000.5390
